# A Predictive Model Based on Machine Learning for the Early Detection of Late-Onset Neonatal Sepsis: Development and Observational Study

**DOI:** 10.2196/15965

**Published:** 2020-07-31

**Authors:** Wongeun Song, Se Young Jung, Hyunyoung Baek, Chang Won Choi, Young Hwa Jung, Sooyoung Yoo

**Affiliations:** 1 Healthcare ICT Research Center Office of eHealth Research and Businesses Seoul National University Bundang Hospital Seongnam-si Republic of Korea; 2 Department of Pediatrics Seoul National University Bundang Hospital Seongnam-si Republic of Korea

**Keywords:** prediction, late-onset neonatal sepsis, machine learning

## Abstract

**Background:**

Neonatal sepsis is associated with most cases of mortalities and morbidities in the neonatal intensive care unit (NICU). Many studies have developed prediction models for the early diagnosis of bloodstream infections in newborns, but there are limitations to data collection and management because these models are based on high-resolution waveform data.

**Objective:**

The aim of this study was to examine the feasibility of a prediction model by using noninvasive vital sign data and machine learning technology.

**Methods:**

We used electronic medical record data in intensive care units published in the Medical Information Mart for Intensive Care III clinical database. The late-onset neonatal sepsis (LONS) prediction algorithm using our proposed forward feature selection technique was based on NICU inpatient data and was designed to detect clinical sepsis 48 hours before occurrence. The performance of this prediction model was evaluated using various feature selection algorithms and machine learning models.

**Results:**

The performance of the LONS prediction model was found to be comparable to that of the prediction models that use invasive data such as high-resolution vital sign data, blood gas estimations, blood cell counts, and pH levels. The area under the receiver operating characteristic curve of the 48-hour prediction model was 0.861 and that of the onset detection model was 0.868. The main features that could be vital candidate markers for clinical neonatal sepsis were blood pressure, oxygen saturation, and body temperature. Feature generation using kurtosis and skewness of the features showed the highest performance.

**Conclusions:**

The findings of our study confirmed that the LONS prediction model based on machine learning can be developed using vital sign data that are regularly measured in clinical settings. Future studies should conduct external validation by using different types of data sets and actual clinical verification of the developed model.

## Introduction

With the developments in the care system of neonate intensive care units (NICUs), the survival rates of very low birth weight infants have greatly increased. However, neonatal sepsis is still associated with most morbidities and mortalities in the NICUs, and 20% of the deaths in infants weighing <1500 g has been reported to be caused by sepsis. Moreover, infants with sepsis are about three times more likely to die compared to those without sepsis [1]. Neonatal sepsis is categorized into early-onset neonatal sepsis occurring within 72 hours of birth and late-onset neonatal sepsis (LONS) occurring between 72 hours and 120 days after birth [1,2]. Early-onset neonatal sepsis is caused by an in utero infection or by vertical bacterial transmission from the mother during vaginal delivery, while LONS is caused not only by vertical bacterial transmission but also by horizontal bacterial transmission from health care providers and the environment.

Sepsis due to group B *Streptococcus*, which is the most common cause of early-onset neonatal sepsis, can be reduced by 80% before delivery, and intrapartum antibiotic prophylaxis is given when necessary. However, in the case of LONS, unlike early-onset neonatal sepsis, there is no specific antibiotic prophylaxis and there is no robust algorithm that can contribute to its early detection in nonsymptomatic newborns [3,4]. A blood culture test is required for the confirmatory diagnosis of LONS, but it takes an average of 2-3 days to obtain blood culture results. Generally, empirical antibiotic treatments are prescribed to reduce the risk of treatment delay. Even if a negative finding is reported for blood culture, antibiotic therapy is prolonged when the clinical symptoms of LONS are manifested because of the possibility of false-negative blood culture results [5,6]. This treatment process results in bacterial resistance, adverse effects due to prolonged antibiotic therapy, and increased medical costs.

Since several studies have analyzed medical imaging data such as computed tomography and magnetic resonance imaging scans and radiographs by using deep learning and machine learning, recent studies have developed prediction models for the early diagnosis of bloodstream infections and symptomatic systemic inflammatory response syndrome in newborns [7-9].

Griffin et al [10,11] presented a method for identifying the early stage of sepsis by checking the abnormal phase of heart rate characteristics. Stanculescu et al [12] applied the autoregressive hidden Markov model to physiological events such as desaturation and bradycardia in infants and predicted the occurrence of an infection by using the onset prediction model. In addition, a model was presented to make predictions by generating a machine learning model based on vital signs or laboratory features recorded in the electronic medical record (EMR) of an infant [13,14]. However, heart rate characteristics can be affected by respiratory deterioration and surgical procedures in addition to sepsis [15] and heart rate characteristics cannot be obtained in patient monitors without an heart rate characteristic index function. The existing prediction models also involved high computational cost, high-resolution data, or laboratory parameters such as complete blood cell count, immature neutrophil to total neutrophil ratio, and polymorphonuclear leukocyte counts.

Studies on machine learning prediction models using EMR data have inherent problems such as high dimensionality and sparsity, data bias, and few abnormal events [16-18]. Previous studies have tried to resolve the abovementioned problems by using several techniques such as oversampling, undersampling, data handling, and feature selection [19-22]. However, the performance of the model that learned processed data by using data augmentation has not significantly improved compared to that of the previous prediction models, and the EMR-based prediction model is still being challenged [17,20,21]. Therefore, by using data from the Medical Information Mart for Intensive Care III (MIMIC-III) database [23], we aimed to apply the feature selection algorithm to develop a machine learning model that reliably predicts LONS by using low sparsity and few scenarios and to examine the feasibility of the developed prediction model by using noninvasive vital sign data and machine learning technology. In addition, we sought to identify clinically significant vital signs and their corresponding feature analysis methods in LONS.

## Methods

### Data Source and Target Population

In this study, the MIMIC-III database [23], which consisted of Beth Israel Deaconess Medical Center’s public data on admission in the intensive care unit, was used as the data source. The use of data from the MIMIC-III database for research was approved by the Institutional Review Boards of Beth Israel Deaconess Medical Center and Massachusetts Institute of Technology. NICU inpatients in the 2001-2008 MIMIC-III database were selected as the total population, and their data were extracted. The patients were assigned to sepsis and control groups. The sepsis group consisted of patients with diagnostic codes of septicemia, infections specific to the perinatal period, sepsis, septic shock, systemic inflammatory response syndrome, etc, based on the discharge report. The diagnostic record of the MIMIC-III database utilized the International Classification of Diseases, Ninth Revision, Clinical Modification codes 038 (septicemia), 771 (infants specific to the perinatal period), 995.9 (systemic inflammatory response syndrome), or 785.52 (septic shock), including the abovementioned diagnosis.

### Identification of the Sepsis Diagnosis Events

Since the diagnosis table of the MIMIC-III database does not contain information on the timing of diagnosis, this information had to be extracted indirectly from the laboratory test order and intervention information to deduce the timing of diagnosis. Generally, positive blood culture results, clinical deterioration, and high C-reactive protein levels are considered as risk factors, and antibiotic treatment is given by aggregating the information on risk factors [3,4,6]. However, in preterm infants, it is difficult to distinguish the normal conditions of the neonatal period from the clinical signs of sepsis, and since the C-reactive protein value could not be obtained from the MIMIC-III database, the timing of sepsis diagnosis was extracted based on the time of blood culture testing and antibiotic prescription. Generally, a positive blood culture result is selected as the gold standard based on the criteria used to confirm sepsis. However, since the amount of blood samples that can be collected from preterm or very low birth weight infants is very limited, the number of blood cultures was also small. Moreover, false-negative results may occur because of the low sensitivity of the blood culture, prior use of broad-spectrum antibiotics, and incubation time of the neonatal blood culture [24,25]. Therefore, the timing of sepsis diagnosis was extracted based on the time of the administration of the order of broad-spectrum antibiotics, time of antibiotic administration through intravenous routes, and the time of blood culture order. In the MIMIC-III database, the date on which the item of SPEC_TYPE_DESC in the MICROBIOLOGY EVENTS table was marked as BLOOD CULTURE was assigned as the date of blood culture and the date on which the DRUG was broad-spectrum antibiotics and the ROUTE was filled as IV was used as the antibiotic date in the PRESCRIPTION table.

### Feature Processing and Imputation

In the machine learning model, the following features were selected: heart rate, systolic blood pressure, diastolic blood pressure, mean blood pressure, oxygen saturation, respiratory rate, and body temperature. In the MIMIC-III database, the vital sign and laboratory data that can be used as the candidate features of the predictive models were heart rate, systolic blood pressure, diastolic blood pressure, mean blood pressure, respiratory rate, body temperature, oxygen saturation, Glasgow Coma Scale score, white blood cell count, red blood cell count, platelet count, bilirubin level, albumin level, pH, potassium level, sodium level, creatinine level, blood urea nitrogen, glucose level, partial pressure of carbon dioxide, fraction of inspired oxygen, serum bicarbonate levels, hematocrit, tidal volume, mean airway pressure, peak airway pressure, plateau airway pressure, and Apgar score. Among them, the primary vital signs (body temperature, heart rate, respiratory rate, and blood pressure) and oxygen saturation levels were recorded periodically, whereas the utilization of the other measured values were limited because they were not recorded periodically or they were recorded only for specific patients. Therefore, body temperature, heart rate, respiratory rate, blood pressure, and oxygen saturation that can be commonly applied in predictive models were selected as the features. Moreover, these vital signs are usually accessible from the bedside, do not involve laboratory tests, and can be applied in most hospitals. However, although the current value of the vital signs can be intuitively used as input data, irregular observation cycles of the patient can increase its complexity. Hence, in this study, we tried to increase the accuracy of the actual physiological deterioration of the patient by additionally calculating the statistical and current values of the vital signs and comparing and evaluating the performance of the significant statistical values and observation period for each vital sign. The statistical values, vital signs, and processed observation window size used for the generation are shown in Table 1. In this study, we used statistical values, which are used by many EMR-based prediction models and time series analysis. However, Fourier transform analysis, wavelet transform, and spectrum analysis, which are mainly applied in time series, were excluded from this study because they require high-frequency and relatively periodic data to produce significant results.

For the normal distribution for goodness of fit test, the Shapiro-Wilk test was used for <5000 samples and the Kolmogorov-Smirnov test was used for ≥5000 samples. These normality tests were used when selecting the suitable statistical method depending on the family of distributions. For the correlation, Pearson’s correlation was used for normally distributed continuous variables; otherwise, Spearman’s correlation was used. Entropy was calculated by estimating the probability density function of the variable with Gaussian kernel density evaluation if a normal distribution was not satisfied. Statistical significance was set to .05. The data quality was assessed by missing value filter and three-sigma rule, and the last observation carried forward method was applied for vital signs assessed as not meeting data quality. The last observation carried forward method is similar to the use of vital signs for diagnosis in general clinical practice and has been mainly used as the imputation method of missing values in clinical prediction models. When there was no measured value, the missing value in the data applied zero imputation to show that it was never measured in the prediction model. Zero imputation was conducted if the calculation could not be performed for reasons such as divided by zero after applying the statistical feature processing.

**Table 1:** Experimental settings of the vital signs, statistical methods, and processed window time. h: hours.

Category	Experimental options
Value of vital signs	Heart rate, respiratory rate, oxygen saturation, systolic blood pressure, mean blood pressure, diastolic blood pressure, body temperature
Statistical method of feature processing	Mean, median, minimum, maximum, standard deviation, skewness, kurtosis, slope, entropy, delta, absolute delta, correlation coefficient, cross-correlation
Processed observation window size	3 h, 6 h, 12 h, 24 h

### Feature Selection Algorithms

**Figure 1:**
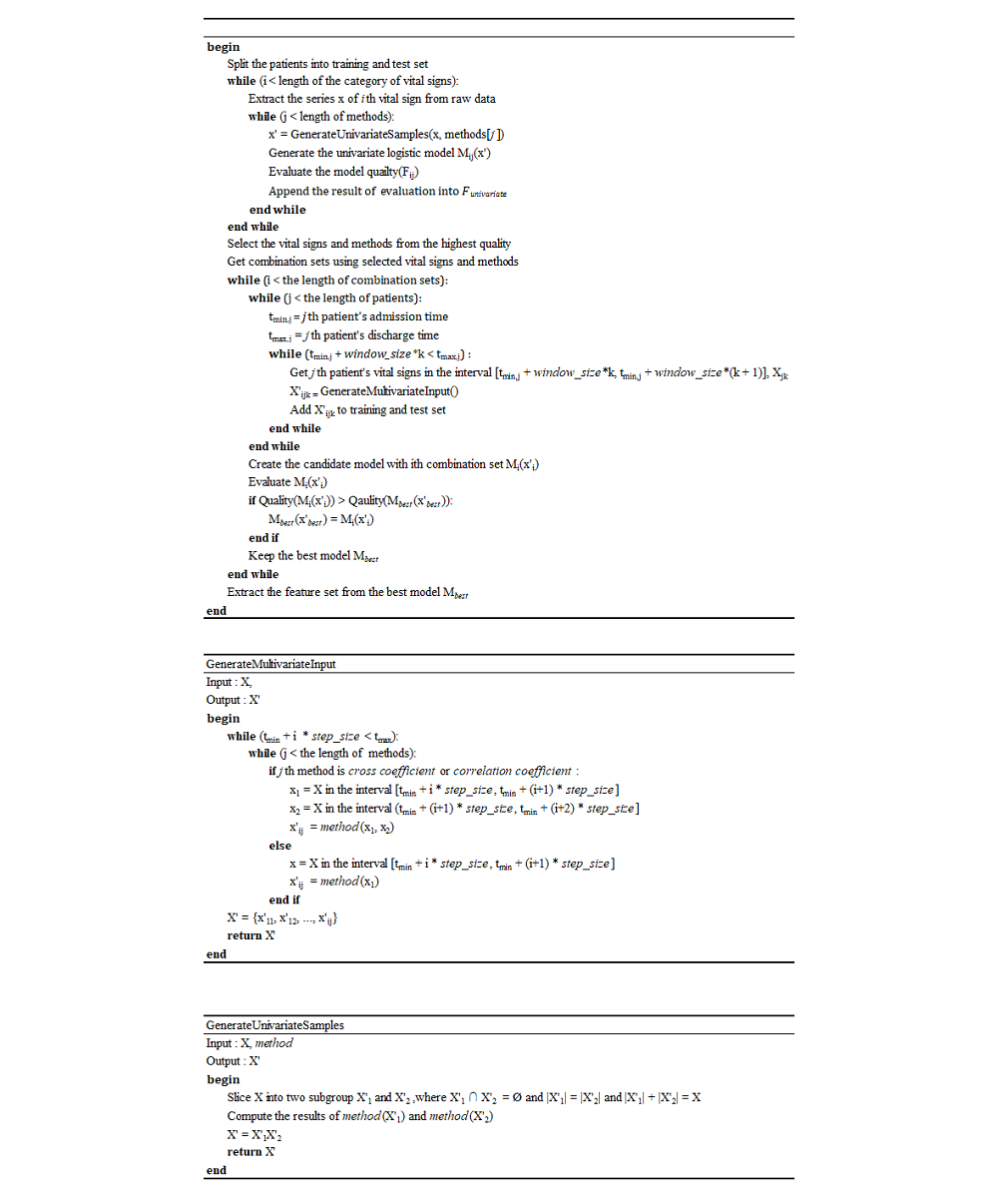
Proposed feature selection algorithm.

To increase the model’s performance and to exclude statistical feature values with low feature importance, a method that has been used and verified mainly in the existing machine learning was used. The feature selection method and algorithm were selected because of the large sparsity of data used in this study and because the coefficient was not larger than that of the typical data (Table 1). In addition, the feature selection algorithm presented in this study was applied (Figure 1).

In this study, M is the prediction model, x is the feature derived from each vital sign, and F is the performance of the model and the sum of the receiver operating characteristic (ROC) and average precision. In the case of the data in this study, it was difficult to measure the performance of the minor class when the incidence ratio was too low. Therefore, the classification performance of the major and minor classes for the model selection was evaluated at the same time as the sum of the average precision and area under the ROC (AUROC) curve. When the features were derived from the vital signs, it was limited to the use of only data obtained from the past observation based on the prediction time to prevent any lookahead due to future observations. In addition, to measure the performance of the proposed feature selection algorithm, we compared the methods usually used from each approach of the feature selection techniques. In the filter approach, chi-squared and mutual information gain were selected. In the embedded approach, lasso linear model L1–based feature selection, extra tree, random forest, and gradient boosting tree–based feature selection were selected. The other principal component analyses were excluded. Principal component analysis is mainly used in a high dimensional space; thus, an additional analysis of the generated features is needed. This means that the direct interpretation power is relatively low in terms of the correlation between the predicted results and the feature importance. In addition, principal component analysis has several disadvantages such as the feature transformation is possible only when all the existing features are contained and high computational cost. Thus, principal component analysis was excluded owing to the above problems. To minimize the differences between the models’ coincidence and temporal characteristics, the observation window and feature processing time stamps were used equally and the model was built without data sampling.

### Machine Learning Algorithms

For the classification algorithm of LONS prediction, logistic regression, Gaussian Naïve Bayes, decision tree, gradient boosting, adaptive boosting, bagging classifier, random forest, and multilayer perceptron were selected and assessed. These machine learning classifiers were mainly used in supervised learning methods such as linear model, naive Bayes, decision tree, ensemble method, and neural network model. In the case of the deep learning model, the performance variation was large depending on the number of layers and the change in the learning rate, and the amount of data was not enough to train the deep learning model. Thus, the deep learning models were excluded. To evaluate the performance between the feature selection model and the proposed algorithm, 10% of the target population was used as the feature selection data set, 80% as the train set, and 10% as the test set to perform a stratified 10-fold cross-validation. Then, 100 turns of bootstrapping were applied to obtain the confidence interval for the 95% section of the performance indicator. The model performance indicator enabled a detailed evaluation of the imbalanced data performance by using indicators such as accuracy, AUROC curve, area under the precision-recall curve (APRC), positive predictive value, negative predictive value, and the harmonic mean of precision and recall (F1 score).

### Data Sampling Algorithm

If the data sampling algorithm is applied to model learning after labeling of the data set, a normal model learning is barely attainable because of the imbalanced and overwhelming data. In this study, undersampling algorithms, oversampling algorithms, and a combination of both oversampling and undersampling algorithms, which are data sampling algorithms, were applied to the training set, and the extent to which the model performance for EMR data set was affected was checked using a test set that was not sampled. The oversampling algorithms used were the synthetic minority oversampling technique (SMOTE) [26], adaptive synthetic sampling method [27], and RandomOverSampler. The undersampling algorithms used were NearMiss [28], RandomUnderSampler, All-K-Nearest-Neighbors [29], and InstanceHardnessThreshold [30]. As for the combination of oversampling and undersampling algorithms, SMOTE + Wilson’s Edited Nearest Neighbor (SMOTEENN) rule [31] and SMOTE + Tomek links [32] were applied.

### Evaluation of the Algorithm

**Figure 2:**
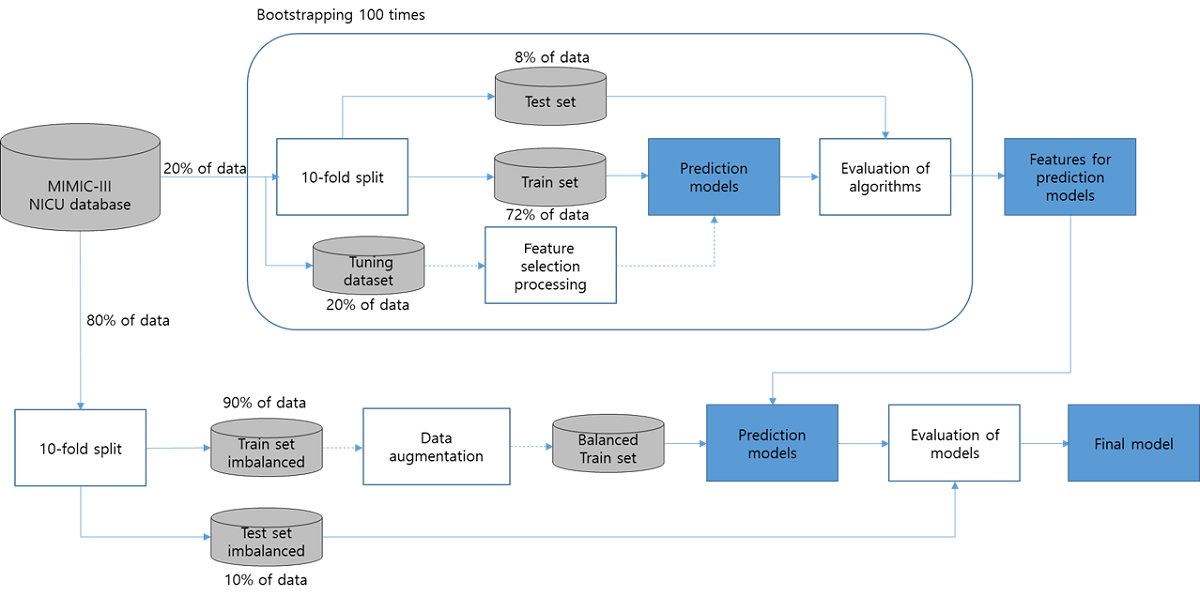
Diagram of the evaluation process for models and algorithms. MIMIC-III: Medical Information Mart for Intensive Care; NICU: neonatal intensive care unit.

The methods presented in Figure 2 were introduced for the evaluation of the feature selection algorithms and prediction model. To prevent leaking of the test set, the MIMIC-III data were divided by organizing the feature selection evaluation data set at 20% and the prediction model evaluation data set at 80% by using a stratified shuffle. To avoid the overestimation in the test set due to the optimized estimator of 10-fold cross-validation, the performance of the prediction models was measured by initializing the hyperparameters at each fold. For the feature selection algorithm, performance was classified based on the Gaussian Naïve Bayesian Classifier as shown in the study by Phyu and Oo [33]. Given that the classifier’s evaluation algorithm is straightforward and that the ensemble classifier such as the gradient-boosted machine can have interactions, nonlinear relationships, and automatically feature selection between features and because there is ambiguity in the statistical properties, the classifier was not selected as the base model [34]. In addition, the mean, minimum, maximum, standard deviation, and median of each vital sign were designated as the baseline features and compared with models that did not perform a feature selection. The existing research model was compared to the model development algorithm presented in this study by presenting both the performance of the presented model and the performance that would have resulted if conducted using the MIMIC-III data. We used Statsmodels and NumPy libraries to analyze the statistical properties. The metric module, a Python module from scikit-learn library, was used to evaluate the classifiers.

## Results

### Characteristics of the Study Population

Table 2 shows the population characteristics of the infants in this study. Of the 7870 infants in the MIMIC-III database, 21 infants were assigned to the clinical LONS group and 2798 infants met the inclusion criteria for the control group. Gestational age, birth weight, and length of stay were significantly different between the clinical LONS and control groups. The median (IQR) gestational age and birth weight in the clinical LONS group were 30 (27.0-34.5) weeks and 0.80 (0.71-1.07) kg, respectively, which were slightly lower than those of the control group whose median (IQR) gestational age and birth weight were 34 (33.5-34.5) weeks and 2.02 (1.58-2.53) kg, respectively. The clinical LONS group showed a significantly longer intensive care unit stay than the control group (87.9 days and 13.3 days, respectively). The male sex rate (%) showed that the male infants in both the clinical LONS and proven sepsis groups had a high risk for infection (61.9% and 51.5%, respectively).

**Table 2:** Characteristics of the target population (N=7870).

Demographic characteristics		NICU^a^, n=96	Clinical LONS^b^ group, n=21	Proven sepsis group, n=715	NICU control group, n=2798
Gestational age (week), median (25th-75th percentile)		34.5 (33.5-35.5)	30 (27.0-34.5)	30 (26.6-34.5)	34 (33.5-34.5)
Birth weight (kg), median (25th-75th percentile)		2.56 (0.36-3.27)	0.80 (0.71-1.07)	0.98 (0.72-1.28)	2.02 (1.58-2.53)
Length of stay (day), median (25th-75th percentile)		0.9 (0.1-10.0)	87.9 (61.9-110.9)	71.2 (42.2-107.2)	13.3 (7.1-28.5)
Mortality in the hospital, n (%)		64 (0.8)	3 (3.1)	1 (5.0)	14 (0.5)
**Gender, n (%)**					
	Male, 4243 (53.9)	54 (56.3)	13 (61.9)	368 (51.5)	1508 (53.9)
	Female, 3627 (46.1)	42 (43.7)	8 (38.1)	347 (48.5)	1290 (46.1)
**Race, n (%)**					
	White, 4764 (60.5)	56 (58.3)	13 (61.9)	463 (64.8)	1747 (62.4)
	African American, 865 (11.0)	14 (14.6)	3 (14.3)	77 (10.8)	301 (10.8)
	Asian, 715 (9.1)	2 (2.1)	0 (0.0)	36 (5.0)	161 (5.8)
	Hispanic, 369 (4.7)	3 (3.1)	1 (4.8)	29 (4.1)	136 (4.9)
	Other, 1157 (14.7)	21 (21.9)	4 (19.0)	110 (15.4)	453 (16.2)
**Hospital admission type, n (%)^c^**					
	Newborn, 7859 (99.9)	95 (99.0)	21 (100.0)	713 (99.7)	2787 (96.4)
	Emergency, 220 (2.8)	22 (22.9)	7 (33.3)	9 (1.3)	87 (3.0)
	Urgent, 23 (0.3)	0 (0.0)	0 (0.0)	1 (0.1)	16 (0.6)
	Elective, 4 (0.1)	0 (0.0)	0 (0.0)	0 (0.0)	2 (0.1)

^a^NICU: neonatal intensive care unit.

^b^LONS: late-onset neonatal sepsis.

^c^allowed to duplicated admission types.

### Performance of the Feature Selection Algorithm

The performances of the proposed feature selection algorithm and the existing feature selection algorithm were compared after 100 turns of bootstrapping; the measured performance by the algorithm is shown in Table 3. Given that the AUROC and accuracy rate are likely to be overestimated in the imbalanced data such as this study’s data, performance was evaluated based on the APRC and F1 measure, which can evaluate the classification performance for major and minor classes. If the window size is 6 hours, the accuracy of the chi-squared feature selection was the highest at 0.60. The extra tree–based feature selection showed a higher performance with AUROC of 0.79, APRC of 0.23, and F1 score of 0.21. When the goal window size was set at 12 hours, the chi-squared (accuracy 0.68, positive predictive value 0.18), extra tree (APRC 0.24), and the proposed algorithm (AUROC 0.79, F1 score 0.25, and weighted-F1 0.65) showed a higher performance than the baseline. However, the feature selection of the manual information gain and lasso L1 penalty classification was still lower than the performance of the baseline model. In a 24-hour window, the proposed algorithm displayed an overall high performance with AUROC of 0.81 (0.81-0.82), APRC of 0.24 (0.23-0.25), and F1 score of 0.33 (0.32-0.34). When the compatibility interval was evaluated, a uniform performance was displayed despite the variations caused by the sample. Overall, as the duration of the observation window increased, the model receiving the features consisting of statistical values as input had improved performance compared to the baseline feature model. The lasso L1 penalty classification model, which is a univariate method, shows the highest indicator with an accuracy of 0.90. However, an AUROC of 0.69 and F1 score of 0.05 indicate that a feature that can barely distinguish normal from suspected infection conditions was selected. The wrapper method feature selection, which was expected to show a high performance, showed a lower performance than the baseline feature model when the observation window was 6 hours. When the observation time was increased to 12 or 24 hours, the extra tree feature selection showed a high performance. However, as the confidence interval appears wider, the robustness based on the sample population changes is lower than those of the other feature selection algorithms. In particular, the feature selection of the feature importance in the random forest and gradient boosting classifier showed an AUROC of 0.56-0.62 and 0.69-0.75, respectively, at 12 hours, and with the 24-hour window, it showed a wide range of confidence intervals at 0.72-0.79 and 0.75-0.81, respectively.

**Table 3:** Comparison results for various feature selection algorithms^a^.

Window size and algorithm		Accuracy^b^, odds ratio (95% CI)		AUROC^c^, odds ratio (95% CI)		APRC^d^, odds ratio (95% CI)		F1^e^, odds ratio (95% CI)		Weighted-F1^f^, odds ratio (95% CI)		PPV^g^, odds ratio (95% CI)		NPV^h^, odds ratio (95% CI)	
**24 hours**															
	Proposed		0.76 (0.75-0.78)		*0.81 (0.80-0.81)*		*0.31(0.31-0.32)*		*0.39 (0.38-0.40)*		0.80 (0.79-0.81)		0.28 (0.27-0.29)		0.95 (0.95-0.96)
	CS^i^		*0.83 (0.81-0.84)*		0.77 (0.76-0.77)		0.28 (0.27-0.29)		0.34 (0.34-0.35)		*0.83 (0.82-0.85)*		*0.30 (0.29-0.31)*		0.92 (0.92-0.93)
	MIG^j^		0.15 (0.13-0.17)		0.53 (0.51-0.54)		0.12 (0.12-0.13)		0.20 (0.20-0.21)		0.08 (0.06-0.11)		0.11 (0.11-0.12)		*0.99 (0.98-0.99)*
	LL1^k^		0.27 (0.23-0.31)		0.54 (0.52-0.55)		0.12 (0.12-0.13)		0.22 (0.21-0.22)		0.26 (0.21-0.30)		0.13 (0.12-0.13)		0.93 (0.93-0.94)
	ET^l^		0.64 (0.60-0.68)		0.79 (0.77-0.81)		0.31 (0.29-0.32)		0.36 (0.35-0.37)		0.68 (0.64-0.73)		0.24 (0.23-0.26)		0.97 (0.96-0.97)
	RF^m^		0.31 (0.27-0.36)		0.65 (0.61-0.68)		0.20 (0.18-0.23)		0.25 (0.24-0.26)		0.30 (0.25-0.36)		0.15 (0.14-0.16)		0.98 (0.98-0.98)
	GB^n^		0.49 (0.44-0.54)		0.72 (0.70-0.75)		0.25 (0.23-0.27)		0.30 (0.29-0.32)		0.51 (0.45-0.57)		0.19 (0.18-0.21)		0.97 (0.97-0.98)
	Baseline		0.56 (0.53-0.58)		0.77 (0.77-0.77)		0.27 (0.26-0.28)		0.30 (0.29-0.31)		0.62 (0.59-0.65)		0.19 (0.18-0.19)		0.96 (0.96-0.97)
**12 hours**															
	Proposed		0.65 (0.62-0.68)		0.75 (0.75-0.76)		0.25 (0.24-0.25)		*0.31 (0.30-0.32)*		0.70 (0.67-0.73)		0.21 (0.20-0.22)		0.95 (0.95-0.95)
	CS		*0.77 (0.75-0.78)*		0.72 (0.71-0.72)		0.22 (0.22-0.23)		0.30 (0.29-0.30)		*0.79 (0.78-0.81)*		*0.23 (0.22-0.23)*		0.93 (0.92-0.93)
	MIG		0.17 (0.15-0.19)		0.58 (0.56-0.60)		0.15 (0.14-0.16)		0.21 (0.20-0.21)		0.13 (0.10-0.16)		0.11 (0.11-0.11)		0.98 (0.97-0.98)
	LL1		0.11 (0.11-0.11)		0.50 (0.50-0.50)		0.11 (0.11-0.11)		0.20 (0.19-0.20)		0.02 (0.02-0.02)		0.11 (0.11-0.11)		*1.00 (1.00-1.00)*
	ET		0.48 (0.44-0.51)		*0.78 (0.77-0.80)*		*0.29 (0.28-0.30)*		0.29 (0.27-0.30)		0.53 (0.49-0.57)		0.17 (0.16-0.19)		0.97 (0.97-0.98)
	RF		0.25 (0.21-0.29)		0.61 (0.58-0.64)		0.18 (0.16-0.19)		0.23 (0.22-0.24)		0.22 (0.17-0.27)		0.13 (0.12-0.14)		0.99 (0.99-0.99)
	GB		0.41 (0.37-0.45)		0.74 (0.72-0.76)		0.24 (0.23-0.26)		0.27 (0.25-0.27)		0.45 (0.40-0.50)		0.16 (0.15-0.17)		0.97 (0.97-0.98)
	Baseline		0.62 (0.59-0.66)		0.73 (0.73-0.74)		0.23 (0.23-0.24)		0.30 (0.30-0.31)		0.67 (0.63-0.71)		0.20 (0.19-0.21)		0.95 (0.95-0.95)
**6 hours**															
	Proposed		0.44 (0.40-0.47)		0.70 (0.70-0.71)		0.20 (0.20-0.21)		0.25 (0.24-0.25)		0.49 (0.45-0.52)		0.15 (0.14-0.16)		0.96 (0.95-0.96)
	CS		0.56 (0.52-0.61)		0.67 (0.65-0.68)		0.18 (0.17-0.19)		0.25 (0.24-0.26)		0.60 (0.56-0.65)		0.16 (0.16-0.17)		0.93 (0.93-0.94)
	MIG		0.11 (0.11-0.11)		0.50 (0.50-0.50)		0.11 (0.11-0.11)		0.19 (0.19-0.20)		0.03 (0.02-0.03)		0.11 (0.11-0.11)		0.99 (0.98-1.00)
	LL1		0.11 (0.11-0.11)		0.50 (0.50-0.50)		0.11 (0.11-0.11)		0.19 (0.19-0.20)		0.02 (0.02-0.02)		0.11 (0.11-0.11)		*1.00 (1.00-1.00)*
	ET		0.46 (0.41-0.50)		0.71 (0.69-0.74)		*0.23 (0.22-0.24)*		0.28 (0.26-0.29)		0.49 (0.43-0.54)		0.17 (0.16-0.18)		0.97 (0.96-0.97)
	RF		0.30 (0.25-0.34)		0.61 (0.59-0.64)		0.17 (0.15-0.18)		0.17 (0.15-0.18)		0.22 (0.21-0.23)		*0.28 (0.22-0.34)*		0.97 (0.96-0.98)
	GB		0.37 (0.32-0.42)		0.66 (0.63-0.69)		0.19 (0.18-0.21)		0.25 (0.24-0.26)		0.38 (0.32-0.44)		0.15 (0.14-0.16)		0.96 (0.95-0.97)
	Baseline		*0.60 (0.56-0.63)*		*0.72 (0.71-0.72)*		0.21 (0.21-0.22)		*0.29 (0.21-0.22)*		*0.65 (0.61-0.69)*		0.18 (0.18-0.19)		0.95 (0.95-0.95)

^a^The highest score in each column is shown in italics.

^b^Accuracy: (true positive + true negative) / (positive + negative).

^c^AUROC: area under the receiver operating characteristic.

^d^APRC: area under the precision recall curve.

^e^F1: harmonic mean of precision and recall.

^f^Weighted-F1: macro F1 measurement.

^g^PPV: positive predictive value.

^h^NPV: negative predictive value.

^i^CS: chi-square test.

^j^MIG: mutual information gain.

^k^LL1: lasso L1 penalty classification.

^l^ET: extra tree.

^m^RF: random forest.

^n^GB: gradient boosting.

### Performance of Data Sampling

Data sampling was measured by fixing the observation time to 24 hours, applying sampling only on training data using the Gaussian Naïve Bayesian classifier and performing stratified 10-fold cross validation. The results of the accuracy analysis showed that the adaptive synthetic sampling method, All-K-Nearest-Neighbors, InstanceHardnessThreshold, and SMOTEENN performed better than the average value of 0.7, which exceeds the 0.579 of the original data. AUROC and APRC showed that all sampling methods, except SMOTEENN, showed a lower or similar performance to the original ones. In the F1 score, SMOTEENN and instance hardness threshold had a higher performance than the original ones.

### Characteristics of the Selected Features

The features obtained from the proposed feature selection method are shown in Table 4. Clinicians might be provided with clinical information on selected features through plots in the form of Table 5 and Multimedia Appendix 1. Table 5 represents the feature importance of the onset after 24 hours calculated by the prediction model learned based on the values of the selected features. Multimedia Appendix 1 provides information on how the prediction model made decisions. Three features were selected among the features mainly selected for each vital sign, and the difference of the latent feature selected based on the window size was confirmed. For the 24-hour window size, the delta between the current and previous measurements was the main variable for all the vital signs. Of these, the kurtosis of the respiratory rate, kurtosis of the body temperature, standard oxygen saturation, and the delta of blood pressures were extracted similarly to the significant feature of the septic shock prediction model [35] for adult patients in the MIMIC-III database, as presented by Carrara et al. As the window size decreased, the data characteristics of the features shifted in importance to mean, entropy, and entropy of delta. This is probably because, in newborns with suspected infection, the frequency of the records increased within the same period such that it affected the entropy increase and was selected as the main variable. When the *P* value of the feature was analyzed using multivariate logistic regression and by focusing on the infection and noninfection points of the statistically significant variables, the oxygen saturation showed desaturation symptoms and wide oxygen saturation changes at the infection point. For the heart rate, tachycardia symptoms were observed at the point of infection. For the body temperature, a delta kurtosis showed a lower expected infection point. Unstable temperature, bradycardia, tachycardia, and hypotension, which are the clinical signs of LONS, were measured [25]. The statistical variable was found to have a lower or similar performance compared to the baseline model for the 12-hour window size. This shows that at least 12 hours of accumulated vital signs must be statistically analyzed so that they can be used as significant physiomarkers.

**Table 4:** Selected features from the proposed feature selection algorithm.

Vital signs and prediction window size		Statistical method of feature processing
**Heart rate**		
	24 hours	Mean, median absolute delta, minimum absolute delta
	12 hours	Mean, minimum absolute delta, median absolute delta
	6 hours	Mean, entropy delta, entropy
**Respiratory rate**		
	24 hours	Mean, median absolute delta, kurtosis absolute delta
	12 hours	Mean, entropy delta, minimum absolute delta
	6 hours	Mean, entropy absolute delta, entropy delta
**Oxygen saturation**		
	24 hours	Mean, standard deviation delta, maximum absolute delta
	12 hours	Mean, maximum absolute delta, standard deviation delta
	6 hours	Mean, entropy delta, entropy absolute delta
**Diastolic blood pressure**		
	24 hours	Mean, maximum absolute delta, maximum delta
	12 hours	Mean, kurtosis delta, kurtosis absolute delta
	6 hours	Mean, entropy delta, entropy absolute delta
**Mean blood pressure**		
	24 hours	Mean, maximum absolute delta, maximum delta
	12 hours	Mean, maximum absolute delta, kurtosis delta
	6 hours	Mean, entropy delta, entropy absolute delta
**Systolic blood pressure**		
	24 hours	Mean, maximum absolute delta, maximum delta
	12 hours	Mean, kurtosis delta, kurtosis absolute delta
	6 hours	Mean, entropy absolute delta, entropy delta
**Body temperature**		
	24 hours	Mean, kurtosis delta, mean absolute delta
	12 hours	Mean, entropy delta, entropy absolute delta
	6 hours	Mean, entropy delta, entropy absolute delta

**Table 5:** An example of the prediction feature importance obtained from the prediction model based on the feature selection algorithm.

Vital signs	Statistical method of feature processing	Feature importance values
Body temperature	Mean	0.282
Oxygen saturation	Mean	0.133
Oxygen saturation	Standard deviation delta	0.126
Heart rate	Mean	0.106
Body temperature	Mean absolute delta	0.052
Heart rate	Median absolute delta	0.046
Respiratory rate	Mean	0.042
Mean blood pressure	Mean	0.032
Body temperature	Kurtosis delta	0.022
Mean blood pressure	Maximum absolute delta	0.022
Diastolic blood pressure	Maximum absolute delta	0.019
Mean blood pressure	Maximum delta	0.018
Respiratory rate	Kurtosis absolute delta	0.017
Systolic blood pressure	Maximum absolute delta	0.016
Diastolic blood pressure	Mean	0.013
Systolic blood pressure	Mean	0.013
Respiratory rate	Median absolute delta	0.011
Oxygen saturation	Maximum absolute delta	0.010
Diastolic blood pressure	Maximum delta	0.009
Systolic blood pressure	Maximum delta	0.006
Heart rate	Minimum absolute delta	0.004

### Performance of the Prediction Model

The models presented in this study and those developed in a previous study are shown in Table 6. The following 2 model types were developed based on the onset point: a prediction model that predicts LONS occurrence 48 hours earlier and a detection model that discovers LONS at the time of measurement. The overall performance of the presented model was higher than that of the model presented in previous studies [12,14]. Compared with the NICU sepsis prediction model of MIMIC-III, which has the same data source, the model developed in this study showed a high performance despite the relatively large number of patients. When comparing the model performance, the gradient boosting of the boost type linking multiple week estimators showed an AUROC of 0.881, APRC of 0.536, and F1 score of 0.625 for the prediction model, while the detection model showed a high performance at an AUROC of 0.877, APRC of 0.567, and F1 score of 0.653. The logistic regression and multilayer perceptron with L2 penalty showed an AUROC of 0.874 and 0.860, APRC of 0.558 and 0.496, and F1 scores of 0.593 and 0.542, respectively, for the prediction model, whereas the detection model showed AUROC of 0.874 and 0.860, APRC of 0.558 and 0.534, and F1 scores of 0.615 and 0.595, respectively, which showed an overall higher performance than the existing LONS prediction models.

**Table 6:** Performance results of the prediction models (microaverage).

Model (Validation data source)		Forecast (h)	Accuracy ^a^	AUROC^b^	APRC^c^	F1^d^	Weighted-F1^e^	PPV^f^	NPV^g^
**Proposed optimization algorithm LONS^h^ prediction model (MIMIC-III)^i^**									
	Logistic regression	48	0.812	0.861	0.446	0.522	0.835	0.395	0.958
	Gaussian Naïve Bayes	48	0.694	0.821	0.394	0.424	0.743	0.283	0.964
	Decision tree classifier	48	0.811	0.841	0.449	0.504	0.833	0.389	0.950
	Extra tree classifier	48	0.867	0.803	0.367	0.131	0.822	0.527	0.874
	Bagging classifier	48	0.863	0.771	0.335	0.251	0.835	0.469	0.883
	Random forest classifier	48	0.867	0.805	0.371	0.205	0.831	0.514	0.879
	AdaBoost^j^ classifier	48	0.825	0.831	0.421	0.507	0.842	0.407	0.944
	Gradient boosting classifier	48	0.845	0.859	0.462	0.522	0.856	0.445	0.939
	Multilayer perceptron classifier	48	0.811	0.841	0.449	0.504	0.833	0.389	0.950
**Proposed optimization algorithm detection model (MIMIC-III)**									
	Logistic regression	0-48	0.798	0.862	0.568	0.619	0.814	0.501	0.943
	Gaussian Naïve Bayes	0-48	0.690	0.806	0.492	0.523	0.720	0.380	0.942
	Decision tree classifier	0-48	0.812	0.614	0.306	0.376	0.786	0.572	0.839
	Extra tree classifier	0-48	0.809	0.794	0.491	0.180	0.748	0.683	0.813
	Bagging classifier	0-48	0.812	0.774	0.461	0.327	0.777	0.592	0.831
	Random forest classifier	0-48	0.817	0.825	0.513	0.302	0.775	0.656	0.827
	AdaBoost classifier	0-48	0.813	0.835	0.513	0.598	0.822	0.529	0.914
	Gradient boosting classifier	0-48	0.830	0.868	0.592	0.624	0.836	0.563	0.919
	Multilayer perceptron classifier	0-48	0.799	0.849	0.558	0.611	0.813	0.502	0.935

^a^Accuracy: (true positive + true negative) / (positive + negative).

^b^AUROC: area under the receiver operating characteristic.

^c^APRC: area under the precision recall curve.

^d^F1: harmonic mean of precision and recall.

^e^Weighted-F1: macro-F1 measurement.

^f^PPV: positive predictive value.

^g^NPV: negative predictive value.

^h^LONS: late-onset neonatal sepsis.

^i^MIMIC-III: Medical Information Mart for Intensive Care III.

^j^AdaBoost: adaptive boosting.

## Discussion

This study showed that when the biosignals recorded in EMR are used to select and learn features based on the presented algorithm, it is possible to produce a model that can predict LONS 48 hours earlier. Our model also showed a higher or similar performance to the high-resolution model of previous studies. The vital sign–based prediction model, which was based on EMR, showed a model performance that exceeded the model that learned based on the laboratory test, which was presented by Mani et al [14]. When compared with the same classifier, the ROC of the prediction model with our random forest algorithm was 0.805, whereas that of the random forest using the laboratory tests of Mani et al [14] was 0.650, with the vital sign–based learning model showing higher performance. Stanculescu et al’s [12] autoregressive hidden Markov model showed an F1 score of 0.690 and APRC of 0.63, which showed higher performance compared to the vital sign–based prediction model that was based on EMR in this study. However, when compared to the detection model, our vital sign–based prediction model that was based on EMR showed a high overall performance. Even if the ROC of the heart rate characteristics was 0.72-0.77, the vital sign–based prediction model recorded in EMR has a higher predictive accuracy than the electrocardiogram-based presentation model [10]. The presented model is expected to show a high contribution even in environments where high-resolution biometric data cannot be collected or where blood culture and laboratory tests cannot be performed regularly. The feature selection presented in this study showed a robust performance compared to the wrapper and embedded method feature selections, which are mainly used in the existing machine learning. Through the selected feature, the main physiomarker can be extracted conversely from EMR. In particular, for preterm infants whose definitions for the normal range of vital signs are insufficient, statistical variables such as biosignal delta and kurtosis over 24 hours can be used as a basis for classifying a patient’s condition. Blood pressure was not used as a key indicator because of the different patient criteria, but it can be used as a major feature by using statistical processing. Moreover, the contribution of the respiratory rate, which was expected to be a key indicator, was low. This is probably because there was a slight change in the respiratory rate of the infants owing to the intervention and ventilation procedures. The correlation coefficient and cross-correlation, which were expected to be important, showed low predictability in low-resolution EMR data. However, they are expected to yield significant results with a high-resolution data set. The vital sign–based prediction model developed in this study has low interpretability, similar to the deep learning and machine learning prediction models in previous studies [12,14]. However, the feature selection presented in this study shows a high performance in linear classifiers such as logistic regression and shows no significant change in performance in other classifiers. If we take advantage of this, applying the feature selection to models such as the fully connected conditional random field and Bayesian inference that have high interpretability can solve the abovementioned problem. Given that the selected feature has dozens of feature spaces, compared to the hundreds of feature spaces in the previous models, simply looking at the model’s input variable will have sufficiently high interpretability.

This study has the following limitations. First, external validation is required because the training and test data sets were created within the MIMIC-III database. N-fold cross validation was performed to reduce data bias as much as possible, but the results may vary depending on the clinician’s recording cycle, pattern, and policy. Therefore, further research requires progress on whether the model generated by the algorithm is equally applicable to the other EMR databases. Second, the limitation about the data extraction was that the prediction model was generated only with noninvasive signs. This was because the number of noninvasive measurements was relatively higher than that of invasive measurements and thus was extracted from most patients. However, an invasive measurement method has the advantage of providing an accurate measurement value; thus, it is performed for patients requiring intensive observation. In future, it is necessary to study whether there is an improvement in performance when the invasive measurement method is applied to the prediction model of this study. Third, infants without infection may have been included in this study or the timing of sepsis onset might not have been recorded correctly. In clinical practice, empirical antibiotic treatment may be administered to noninfected infants with symptoms of sepsis to reduce mortality. Therefore, there is a limitation that false-positive sepsis can occur. In addition, since the MIMIC-III database covers the period from 2001 to 2008, the data may differ by patient population, treatment, and sepsis definition. Fourth, in the vital sign–based prediction model developed in this study, only multilayer perceptron was applied as a deep learning model. In addition, the performance presented in this study is likely to be lower than the maximum performance that can be modeled because the vital sign–based prediction model that was based on EMR developed in this study is a default model with no hyperparameter tuning. Therefore, advanced deep learning models should be applied to develop sophisticated and accurate prediction models in future studies. Lastly, our model could not be compared with the risk score model and the medical guidelines used in clinical practice. In clinical practice, the results of the hematology tests such as complete blood cell count, immature neutrophil to total neutrophil ratio, and polymorphonuclear leukocyte counts are mainly applied. In the MIMIC-III database used in this study, there was not enough data to record the results of the hematology test as a score model, which makes it difficult to directly compare the performance with the prediction model of the study. Further, ethnicity, gender, and immaturity might affect the outcomes since each factor affects the incidence of sepsis. Previous studies have shown that low birth weight and male gender as risk factors of infection could affect the probability of bloodstream infection. Ethnicity did not seem to directly affect the incidence of sepsis, but the sepsis incidence is different according to the community income level. Therefore, if the aforementioned characteristics of the infants are different from the population of this study, then there is a possibility of obtaining different results. Moreover, the MIMIC-III database lacks the number of infant samples that can be configured for each condition, and it is difficult to show the difference in the results. Nevertheless, acceptable results will be obtained again if the proposed algorithm is reperformed for a specific population. In addition, although the gene type was not recorded in the MIMIC-III database and could not be included, research on gene types should be conducted in the future. If the vital sign–based prediction model that was based on EMR developed in this study is applied to clinical sites, patients with a high LONS risk can be identified up to 48 hours in advance with high accuracy based on the nonregular charts. This could be the basis for triage of patients with a high LONS risk. Combining the predicted results of this algorithm with vital signs traditionally used in clinical sites and test results will help clinicians reach an augmented decision.

In conclusion, we developed a prediction model after generating a key feature with feature selection presented in the EMR data. By doing so, a vital sign–based prediction model that was based on EMR achieved a high prediction performance and robustness compared to the previous feature selection. This research model is expected to significantly reduce the mortality of patients with LONS, and sophisticated predictions can be made through the deep learning model and model optimization. However, the limitations of data extraction and the need to construct a data collection environment remain as the major challenges in applying predictive models in clinical practice. Thus, further research is needed to address these problems.

## Appendix

Multimedia Appendix 1 An example of the decision tree graph for the decision tree classifier.The colors indicate the major class in each node.

## Abbreviations

APRC: area under the precision-recall curve
AUROC: area under the receiver operating characteristic
EMR: electronic medical record
LONS: late-onset neonatal sepsis
MIMIC-III: Medical Information Mart for Intensive Care III
NICU: neonatal intensive care unit
ROC: receiver operating characteristic
SMOTE: synthetic minority oversampling technique
SMOTEENN: SMOTE + Wilson’s Edited Nearest Neighbor Rule
